# Association of age, BMI and their interaction with thyroid nodules in patients with type 2 diabetes mellitus: a cross-sectional study

**DOI:** 10.3389/fendo.2026.1863519

**Published:** 2026-07-01

**Authors:** Xiaoyu Pan, Ruixia Liu, Yi Yao, Yueyue Zhu, Tianrong Pan

**Affiliations:** Department of Endocrinology, The Second Affiliated Hospital of Anhui Medical University, Hefei, Anhui, China

**Keywords:** age, body mass index, interaction, thyroid nodules, type 2 diabetes mellitus

## Abstract

**Background:**

Type 2 diabetes mellitus (T2DM) and thyroid nodules are both prevalent endocrine conditions; the prevalence of comorbidity between the two continues to rise. However, the independent and interactive effects of age and body mass index (BMI) on the development of thyroid nodules in patients with T2DM remain unclear. The objective of this study is to examine the correlation between age, BMI, and their interaction with the risk of developing thyroid nodules in patients with T2DM.

**Methods:**

A retrospective study encompassed 720 hospitalised T2DM patients. The patients were divided into two groups based on the findings of thyroid ultrasound scans: a thyroid nodule group (n=599) and a non-thyroid nodule group (n=121). A comparison was made between the baseline clinical characteristics of the two groups. Univariate and multivariate logistic regression analyses were performed to identify independent risk factors for thyroid nodules, and stratified analyses and interaction tests were conducted to assess the interaction between age and BMI.

**Results:**

Multivariate logistic regression analysis showed that age was an independent risk factor for thyroid nodules in patients with T2DM (P<0.001), and the risk of thyroid nodules in patients aged 45–60 years and ≥60 years was 5.12 times and 9.07 times that of patients aged <45 years, respectively. BMI was an independent protective factor (P<0.05), and the risk of thyroid nodules in patients with BMI 24–28 kg/m² and ≥28 kg/m² was 0.52 times and 0.46 times that of patients with BMI <24 kg/m², respectively. There was a significant interaction between age and BMI (OR = 1.26, 95% CI: 1.08-1.47, P = 0.003). Stratified analysis showed that in the subgroup with BMI ≥24 kg/m², the promoting effect of age on thyroid nodules was more obvious; in the subgroup aged <45 years, the protective effect of BMI was more prominent; in the subgroup aged 45–60 years, BMI ≥28 kg/m² became a risk factor (OR = 8.30, 95% CI: 1.42-48.56, P = 0.019).

**Conclusions:**

Age and BMI have independent and interactive effects on the risk of thyroid nodules in hospitalised T2DM patients. However, the findings may not be generalizable to community-dwelling or outpatient T2DM patients without further validation.

## 1 Introduction

Type 2 diabetes mellitus (T2DM) is a chronic metabolic disorder that has a high prevalence worldwide. The core pathological features of the condition under investigation are insulin resistance and defects in pancreatic β-cell function. In recent years, there has been a demonstrable upward trend in the prevalence of this condition, which has led to its categorisation as a major public health issue (1). Thyroid nodules represent the most prevalent morphological abnormalities of the thyroid gland. As a result of the extensive utilisation of ultrasound procedures, the incidence of thyroid nodules in adult populations has been reported to be between 20% and 76% (2–4). A proportion of these nodules have been observed to harbour a potential for malignancy, thereby constituting a significant health hazard for patients.

Recent studies have found that the prevalence of thyroid nodules among patients with T2DM is significantly higher than in the general population, suggesting a close intrinsic link between glucose metabolism disorders and structural damage to the thyroid (5–7). As established demographic and anthropometric indicators, age and BMI have been demonstrated to be closely associated with the development and progression of T2DM and thyroid nodules. It has been established through previous studies that advanced age is a consistent risk factor for thyroid nodules. As age increases, degenerative changes in thyroid follicular cells and prolonged metabolic stress may elevate the risk of nodule formation (8, 9). However, the conclusions drawn concerning the relationship between BMI and thyroid nodules remain controversial. Some studies suggest that obesity may lead to an increased thyroid volume and the risk of nodule formation through mechanisms such as influencing thyroid hormone secretion and promoting inflammatory responses (10). In contrast, other studies indicate that there is no significant association between BMI and thyroid nodules (11). The observed discrepancies may be attributable to three factors. Firstly, heterogeneity in the study populations may be a contributing factor. Secondly, inadequate control of confounding factors may be another potential explanation. Thirdly, insufficient assessment of the interaction between age and BMI may be a third contributing factor.

At present, there is a paucity of studies that have examined the independent effects of age and BMI on thyroid nodules, as well as their interactive effects, in hospitalised T2DM patients. Consequently, it is challenging to elucidate the combined effect of these factors on the risk of thyroid nodules in this population. Consequently, this retrospective study enrolled 720 hospitalised T2DM patients. The present study set out to ascertain the association between age, BMI, and their interaction with the risk of thyroid nodules in hospitalised T2DM patients. To this end, a comparative analysis was conducted on the baseline characteristics of the thyroid nodule group and the non-nodule group. Logistic regression and stratified analysis were utilised in order to achieve this objective. This provides potential clinical evidence for risk stratification, targeted screening and clinical intervention regarding thyroid nodules in hospitalised T2DM patients.

## 2 Methods

### 2.1 Research subjects

The present study adopts a cross-sectional research design. The study population comprised inpatients from the Department of Endocrinology at the Second Affiliated Hospital of Anhui Medical University, with the study period running from October 2025 to April 2026. A total of 1,035 inpatients were initially enrolled in the study; following screening against strict inclusion and exclusion criteria, 720 patients with T2DM were ultimately included in the study. This study was approved by the Medical Ethics Committee of the Second Affiliated Hospital of Anhui Medical University (Approval No: YX2025-103), and all patients provided written informed consent.

The inclusion criteria for the study are as follows: A confirmed diagnosis of T2DM, in accordance with the relevant criteria set out in the Chinese Guidelines for the Prevention and Treatment of T2DM; aged ≥ 18 years; possession of complete clinical records, with access to thyroid ultrasound results and relevant laboratory test results; voluntary participation in this study and signing of an informed consent form. Exclusion criteria: Patients without T2DM; subjects below 18 years of age; subjects who have not undergone ultrasound scanning for the thyroid region; subjects with a documented medical history of undergoing thyroid surgery, or who have received radioactive iodine therapy; subjects with missing key clinical and/or laboratory test results; subjects with a documented medical history of severe organic diseases such as severe hepatic or renal insufficiency or heart failure; subjects who are pregnant or breastfeeding.

The participants of the study were divided into two groups based on the results of thyroid ultrasound examinations; those with thyroid nodules and those without. Participants from both groups completed the collection of baseline data and testing of relevant indicators.

### 2.2 Baseline data collection

A standardised questionnaire and medical record review were used to collect baseline data from all study participants, including age, gender, smoking history and alcohol consumption history. The presence of comorbidities, including hypertension, metabolic dysfunction-associated steatotic liver disease (MASLD), heart disease and stroke, was determined based on clinically confirmed diagnoses. Ages are categorised based on the universal criteria for young and middle-aged, middle-aged and older, and older adults, into three groups: <45 years, 45–60 years, and ≥60 years.

### 2.3 Medical examination

Physical examinations of the study participants will be conducted by professional healthcare staff in accordance with standardised procedures, and the following parameters will be measured: height, weight, systolic blood pressure (SBP) and diastolic blood pressure (DBP). The height of the subjects was recorded to the nearest 0.1 centimetre, and the weight to the nearest 0.1 kilogram. The body mass index (BMI) was calculated using the following formula: BMI = weight (kg)/height² (m²). The subjects were then categorised into three groups according to the Chinese adult BMI classification criteria. The weight categories employed for the purposes of this study are as follows: less than 24 kg/m², 24–28 kg/m², and greater than 28 kg/m². The measurement of blood pressure is undertaken using a standard mercury sphygmomanometer. Participants are requested to rest quietly for a period of 15 minutes prior to the commencement of the measurement. The process of measuring blood pressure involves the taking of three consecutive measurements at five-minute intervals. The average of these three readings is then taken as the final blood pressure value.

### 2.4 Laboratory testing

Blood samples were collected from the antecubital vein of all study participants in the early morning whilst fasting. Following centrifugation to separate the serum, the relevant laboratory parameters were measured using a fully automated biochemical analyser. These included glycated haemoglobin (HbA1c), fasting blood glucose (FBG), total cholesterol (TC), triglycerides (TG), high-density lipoprotein cholesterol (HDL-C), low-density lipoprotein cholesterol (LDL-C), alanine aminotransferase (ALT), aspartate transaminase (AST), serum creatinine (Scr), uric acid (UA), blood urea nitrogen (BUN), and the calculation of estimated glomerular filtration rate (eGFR). All testing procedures are strictly adhered to in accordance with the standard operating procedures of the laboratory, thus ensuring the accuracy and reliability of the ensuing test results.

### 2.5 Thyroid ultrasound

The thyroid ultrasound scan will be performed by a radiologist with expertise in this field, utilising a colour Doppler ultrasound scanner. The examination encompasses both lobes of the thyroid and the isthmus, encompassing the assessment of size, shape, echogenicity, and the presence of nodules. The following diagnostic criteria have been established for thyroid nodules: A space-occupying lesion that is visible on ultrasound images and exhibits echogenicity distinct from the surrounding tissue, with clear or indistinct margins and a defined shape and size. Benign lesions, such as thyroid cysts and inflammatory nodules, are excluded. The ultrasound findings were independently assessed by two radiologists; in cases of disagreement, a consensus was reached through joint discussion.

### 2.6 Statistical analysis

The analysis was conducted utilising the R software version 4.2.1. Continuous variables that followed a normal distribution are expressed as mean ± standard deviation ( $\overset{—}{x}$ ± s), and comparisons between groups were performed using an independent samples t-test; continuous variables that did not follow a normal distribution are expressed as median (interquartile range), and comparisons between groups were performed using non-parametric tests. Categorical variables are expressed as frequency (%), and comparisons between groups were performed using the chi-square test. Univariate logistic regression analysis was used to identify potential risk factors for thyroid nodules. Variables with P < 0.1 in the univariate analysis were included in the multivariate logistic regression analysis to calculate odds ratios (OR) and 95% confidence intervals (95% CI), thereby identifying independent risk factors. The interaction between age and BMI was assessed by incorporating the interaction term BMI × age in the regression model. The interaction term is defined as Age_cent × BMI_cent. Multivariate logistic regression analyses were conducted stratified by BMI (<24 kg/m², 24–28 kg/m², ≥28 kg/m²) and age (<45 years, 45–60 years, ≥60 years) to further validate the interaction effect between the two factors. A two-sided P-value less than 0.05 was considered to be statistically significant.

## 3 Results

### 3.1 Selection of study participants and baseline characteristics

**Figure 1:**
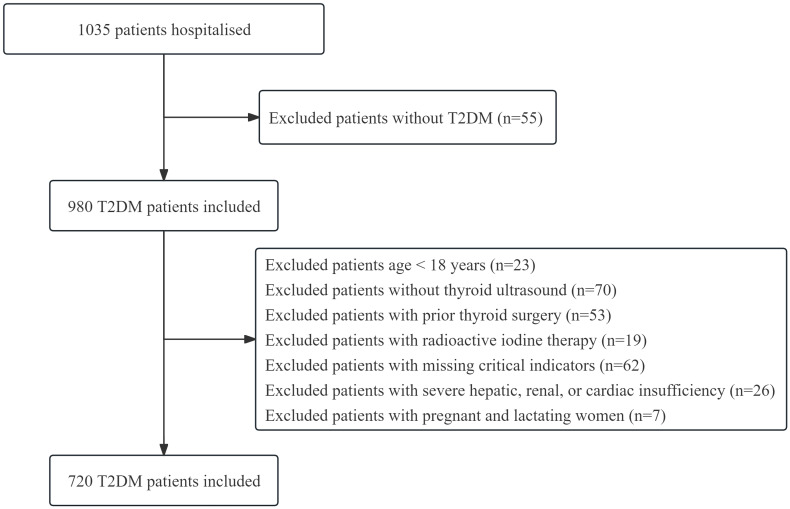
Flowchart of the population included in our study.

The present study initially enrolled 1,035 in-hospital patients. Following the exclusion of patients with non-T2DM, those aged under 18 years, those who had not undergone thyroid ultrasound, those with a history of thyroid surgery or radioiodine therapy, those with missing key indicators, and those with severe cardiac, hepatic or renal impairment, as well as pregnant or breastfeeding women, a total of 720 patients with T2DM were ultimately included in the study. The patients were divided into two groups based on thyroid ultrasound findings: a thyroid nodule group (n = 599) and a group without thyroid nodules (n = 121) (**Figure 1**).

In comparison with the group exhibiting no thyroid nodules, the group with thyroid nodules demonstrated a higher mean age (P < 0.001), lower BMI (P = 0.010), lower DBP (P = 0.026), lower TC levels (P = 0.046), and lower AST and ALT levels (both P < 0.01). The nodule group exhibited a higher proportion of women (P = 0.004), lower rates of smoking, alcohol consumption and MASLD (all P < 0.05), and a higher proportion with a history of stroke (P = 0.004). Statistically significant differences were absent between the two groups in terms of SBP, HbA1c, FBG, HDL-C, LDL-C, TG, BUN, Scr, or history of hypertension and heart disease (all P > 0.05) (**Table 1**).

**Table 1:** Comparison of baseline clinical characteristics in T2DM patients.

Variables	Total (n = 720)	Non-thyroid nodule (n = 121)	Thyroid nodule (n = 599)	*P*
Age (y)	58.09 ± 13.68	49.54 ± 13.89	59.81 ± 12.98	<0.001
BMI (kg/m^2^)	25.14 ± 3.98	25.99 ± 4.19	24.97 ± 3.92	0.010
SBP (mmHg)	130.54 ± 18.38	130.16 ± 16.77	130.62 ± 18.70	0.802
DBP (mmHg)	78.91 ± 11.88	81.10 ± 11.44	78.47 ± 11.92	0.026
HbA1c (%)	8.40 ± 2.01	8.31 ± 1.99	8.41 ± 2.01	0.613
FBG (mmol/L)	7.39 ± 2.93	7.25 ± 2.50	7.42 ± 3.01	0.560
TC (mmol/L)	4.62 ± 1.39	4.85 ± 1.43	4.57 ± 1.38	0.046
TG (mmol/L)	2.08 ± 2.55	2.50 ± 3.05	2.00 ± 2.43	0.050
LDL-C (mmol/L)	2.76 ± 0.96	2.83 ± 1.05	2.75 ± 0.94	0.423
HDL-C (mmol/L)	1.12 ± 0.34	1.08 ± 0.34	1.12 ± 0.33	0.214
ALT (U/L)	25.03 ± 16.77	29.84 ± 19.58	24.04 ± 15.98	0.003
AST (U/L)	22.90 ± 10.77	26.01 ± 13.83	22.27 ± 9.93	0.005
Scr (mmol/L)	68.77 ± 56.00	62.45 ± 32.22	70.06 ± 59.64	0.174
UA (mmol/L)	314.15 ± 97.29	311.74 ± 90.43	314.64 ± 98.70	0.765
BUN (mmol/L)	6.99 ± 4.02	6.58 ± 5.33	7.07 ± 3.69	0.229
eGFR (mL/min/1.73m²)	128.43 ± 48.27	136.56 ± 54.25	126.65 ± 46.73	0.053
Gender, n(%)				0.004
Female	384 (53.33)	50 (41.32)	334 (55.76)	
Male	336 (46.67)	71 (58.68)	265 (44.24)	
Smoking, n(%)	188 (26.11)	44 (36.36)	144 (24.04)	0.005
Drinking, n(%)	228 (31.67)	53 (43.80)	175 (29.22)	0.002
Hyptension, n(%)	393 (54.58)	57 (47.11)	336 (56.09)	0.070
MASLD, n(%)	245 (34.03)	52 (42.98)	193 (32.22)	0.023
Heart disease, n(%)	73 (10.14)	8 (6.61)	65 (10.85)	0.159
Stroke, n(%)	101 (14.03)	7 (5.79)	94 (15.69)	0.004
Age, n(%)				<0.001
< 45	123 (17.08)	48 (39.67)	75 (12.52)	
45-60	221 (30.69)	37 (30.58)	184 (30.72)	
≥ 60	376 (52.22)	36 (29.75)	340 (56.76)	
BMI, n(%)				0.011
< 24	288 (40.00)	34 (28.10)	254 (42.40)	
24-28	295 (40.97)	57 (47.11)	238 (39.73)	
≥ 28	137 (19.03)	30 (24.79)	107 (17.86)	

BMI, body mass index; SBP, systolic blood pressure; DBP, diastolic blood pressure; HbA1c, glycated hemoglobin; BUN, blood urea nitrogen; Scr, scrum creatinine; UA, uric acid; TC, total cholesterol; TG, triglyceride; HDL-C, high-density lipoprotein cholesterol; LDL-C, low-density lipoprotein cholesterol; FBG, fasting blood glucose; ALT, alanine aminotransferase; AST, aspartate transaminase; MASLD, metabolic dysfunction-associated steatotic liver disease; eGFR, estimated Glomerular Filtration Rate.

### 3.2 Analysis of the correlation between BMI and age stratification and the distribution of thyroid nodules

**Figure 2:**
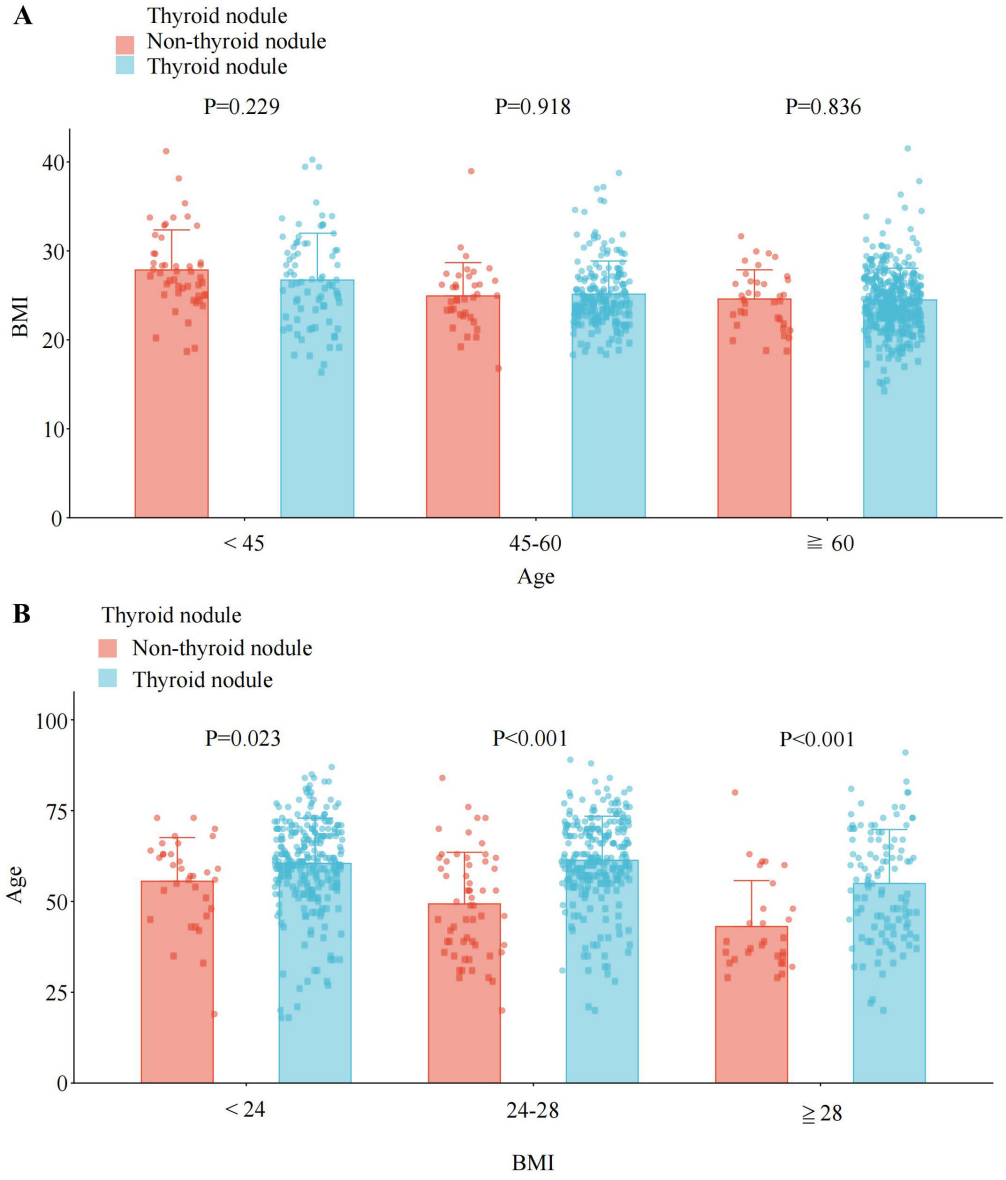
Distribution of age and BMI between the thyroid nodule group and non-thyroid nodule group, and comparison between groups. **(A)** Participants were divided into subgroups based on age grouping. **(B)** Participants were divided into subgroups based on BMI grouping.

A study was conducted in order to analyse differences in distribution between patients with thyroid nodules and those without, across different age ranges and BMI categories. In the age-stratified analysis, P-values of 0.229, 0.918 and 0.836 were obtained for between-group comparisons in each respective subgroup, indicating an absence of statistically significant differences in the distribution of thyroid nodules between the study groups, categorised by age. In the BMI stratification analysis, the P-values for the differences in distribution between the groups were found to be 0.023, <0.001, and <0.001. It is important to note that these values were all found to be statistically significant (**Figure 2**).

### 3.3 Univariate logistic regression analysis

Univariate logistic regression analysis revealed that age, sex, BMI, DBP, ALT, AST, smoking, alcohol consumption, MASLD and stroke were potential risk factors for thyroid nodules in patients with T2DM (all P < 0.05). Among these, advanced age (OR = 1.05, 95% CI: 1.04-1.07, P < 0.001) and stroke (OR = 3.03, 95% CI: 1.37-6.71, P = 0.006) were identified as risk factors for thyroid nodules; high BMI (OR = 0.94, 95% CI: 0.90-0.99, P = 0.010), male gender (OR = 0.56, 95% CI: 0.38-0.83, P = 0.004), smoking (OR = 0.55, 95% CI: 0.37-0.84, P = 0.005), alcohol consumption (OR = 0.53, 95% CI: 0.35-0.79, P = 0.002), MASLD (OR = 0.63, 95% CI: 0.42-0.94, P = 0.023), high DBP (OR = 0.98, 95% CI: 0.97-0.99, P = 0.027), high ALT (OR = 0.98, 95% CI: 0.97-0.99, P < 0.001), high AST (OR = 0.97, 95% CI: 0.96-0.99, P < 0.001) were protective factors. The interaction between age and BMI was statistically significant (OR = 1.26, 95% CI: 1.08-1.47, P = 0.003) (**Table 2**).

**Table 2:** Univariate logistic regression analysis of thyroid nodule in T2DM patients.

Variables	β	S.E	Z	*P*	OR (95%CI)
Age (y)	0.05	0.01	7.16	<0.001	1.05 (1.04-1.07)
BMI (kg/m^2^)	-0.06	0.02	-2.56	0.010	0.94 (0.90-0.99)
SBP (mmHg)	0.00	0.01	0.25	0.801	1.00 (0.99-1.01)
DBP (mmHg)	-0.02	0.01	-2.21	0.027	0.98 (0.97-0.99)
HbA1c (%)	0.03	0.05	0.51	0.612	1.03 (0.93-1.14)
FBG (mmol/L)	0.02	0.04	0.58	0.560	1.02 (0.95-1.10)
TC (mmol/L)	-0.13	0.07	-1.95	0.051	0.88 (0.77-1.00)
TG (mmol/L)	-0.06	0.03	-1.84	0.066	0.94 (0.89-1.00)
LDL-C (mmol/L)	-0.08	0.10	-0.80	0.422	0.92 (0.75-1.13)
HDL-C (mmol/L)	0.40	0.32	1.25	0.213	1.49 (0.80-2.78)
ALT (U/L)	-0.02	0.01	-3.39	<0.001	0.98 (0.97-0.99)
AST (U/L)	-0.03	0.01	-3.35	<0.001	0.97 (0.96-0.99)
Scr (mmol/L)	0.00	0.00	1.37	0.170	1.00 (1.00-1.01)
UA (mmol/L)	0.00	0.00	0.30	0.764	1.00 (1.00-1.00)
BUN (mmol/L)	0.04	0.03	1.20	0.230	1.04 (0.97-1.11)
eGFR (mL/min/1.73m²)	-0.00	0.00	-1.92	0.055	1.00 (0.99-1.00)
Gender, n(%)					
Female					1.00 (Reference)
Male	-0.58	0.20	-2.88	0.004	0.56 (0.38-0.83)
Smoking, n(%)	-0.59	0.21	-2.79	0.005	0.55 (0.37-0.84)
Drinking, n(%)	-0.64	0.20	-3.12	0.002	0.53 (0.35-0.79)
Hyptension, n(%)	0.36	0.20	1.81	0.071	1.43 (0.97-2.12)
MASLD, n(%)	-0.46	0.20	-2.27	0.023	0.63 (0.42-0.94)
Heart disease, n(%)	0.54	0.39	1.39	0.163	1.72 (0.80-3.68)
Stroke, n(%)	1.11	0.41	2.74	0.006	3.03 (1.37-6.71)
BMI × age interaction term	0.23	0.08	2.98	0.003	1.26 (1.08-1.47)

BMI, body mass index; SBP, systolic blood pressure; DBP, diastolic blood pressure; HbA1c, glycated hemoglobin; BUN, blood urea nitrogen; Scr, scrum creatinine; UA, uric acid; TC, total cholesterol; TG, triglyceride; HDL-C, high-density lipoprotein cholesterol; LDL-C, low-density lipoprotein cholesterol; FBG, fasting blood glucose; ALT, alanine aminotransferase; AST, aspartate transaminase; MASLD, metabolic dysfunction-associated steatotic liver disease; eGFR, estimated Glomerular Filtration Rate.

### 3.4 Multivariate analysis of age and thyroid nodules

Utilising a reference group of individuals below the age of 45 years, it was observed that age persisted as an independent risk factor for thyroid nodules following multivariable adjustment. The fully adjusted model demonstrated that the risk of nodules in the 45–60 age group was 5.12 times that of the reference group (95% CI: 2.58-10.15, P < 0.001), while the risk in the ≥ 60 age group increased to 9.07 times (95% CI: 4.17-19.72, P < 0.001). These trends were consistent across all adjusted models (**Table 3**).

**Table 3:** Multivariate logistic regression analyses of age and thyroid nodule.

Variables	Model1		Model2		Model3		Model4	
	OR (95%CI)	*P*	OR (95%CI)	*P*	OR (95%CI)	*P*	OR (95%CI)	*P*
Age								
< 45	1.00 (Reference)		1.00 (Reference)		1.00 (Reference)		1.00 (Reference)	
45-60	3.18 (1.92-5.28)	<0.001	3.21 (1.90-5.41)	<0.001	3.13 (1.82-5.39)	<0.001	5.12 (2.58-10.15)	<0.001
≥ 60	6.04 (3.67-9.96)	<0.001	5.42 (3.22-9.13)	<0.001	4.77 (2.67-8.50)	<0.001	9.07 (4.17-19.72)	<0.001

Model1: Crude; Model2: Adjust: Gender, smoking, drinking and BMI; Model3: Adjust: Gender, smoking, drinking, BMI, hyptension, MASLD, heart disease and stroke; Model4: Adjust: Gender, smoking, drinking, BMI, hyptension, MASLD, heart disease, stroke, HbA1c, FBG, TC, TG, LDL, HDL, ALT, AST, Scr, UA, BUN and eGFR.

### 3.5 Multivariate analysis of BMI and thyroid nodules

Utilising a BMI < 24 kg/m² as the reference group, an elevated BMI was found to be inversely associated with the risk of thyroid nodules following adjustment for multiple factors. The fully adjusted model demonstrated that the risk observed in the BMI 24–28 kg/m² group was 0.52 times that of the reference group (95% CI: 0.30-0.91, P = 0.022), and 0.46 times that of the reference group in the BMI ≥ 28 kg/m² group (95% CI: 0.24-0.91, P = 0.024). These findings indicate that BMI functions as an independent protective factor (**Table 4**).

**Table 4:** Multivariate logistic regression analyses of BMI and thyroid nodule.

Variables	Model1		Model2		Model3		Model4	
	OR (95%CI)	*P*	OR (95%CI)	*P*	OR (95%CI)	*P*	OR (95%CI)	*P*
BMI								
< 24	1.00 (Reference)		1.00 (Reference)		1.00 (Reference)		1.00 (Reference)	
24-28	0.56 (0.35-0.89)	0.013	0.58 (0.36-0.92)	0.020	0.56 (0.34-0.90)	0.016	0.52 (0.30-0.91)	0.022
≥ 28	0.48 (0.28-0.82)	0.007	0.51 (0.29-0.88)	0.015	0.53 (0.30-0.94)	0.029	0.46 (0.24-0.91)	0.024

Model1: Crude; Model2: Adjust: Gender, smoking, drinking and age; Model3: Adjust: Gender, smoking, drinking, age, hyptension, MASLD, heart disease and stroke; Model4: Adjust: Gender, smoking, drinking, age, hyptension, MASLD, heart disease, stroke, HbA1c, FBG, TC, TG, LDL, HDL, ALT, AST, Scr, UA, BUN and eGFR.

### 3.6 The interaction between age and BMI on thyroid nodules

The investigation revealed that when the data were stratified by BMI, no statistically significant differences in the risk of nodules were observed across age groups (all P > 0.05) when BMI was less than 24 kg/m². However, when BMI was between 24 and 28 kg/m², the risk was found to be significantly elevated in the 45–60 and ≥ 60 age groups. Following full adjustment, the odds ratio for the ≥ 60 age group was 18.82 (95% CI: 4.95-71.54, P < 0.001). When BMI was equal to or more than 28 kg/m², the risk was significantly elevated in the 45–60 age group, while it was marginally significant in those aged ≥ 60 (P = 0.055) (**Table 5**).

**Table 5:** Multivariate logistic regression of age and thyroid nodules by BMI stratification.

Variables	Model1		Model2		Model3		Model4	
	OR (95%CI)	*P*	OR (95%CI)	*P*	OR (95%CI)	*P*	OR (95%CI)	*P*
BMI<24								
Age								
< 45	1.00 (Reference)		1.00 (Reference)		1.00 (Reference)		1.00 (Reference)	
45-60	1.47 (0.51-4.26)	0.476	1.40 (0.46-4.26)	0.549	1.39 (0.44-4.42)	0.580	0.78 (0.13-4.58)	0.780
≥ 60	2.83 (0.99-8.11)	0.052	2.67 (0.91-7.78)	0.073	2.70 (0.83-8.77)	0.099	3.72 (0.56-24.66)	0.173
28>BMI≥24								
Age								
< 45	1.00 (Reference)		1.00 (Reference)		1.00 (Reference)		1.00 (Reference)	
45-60	3.68 (1.70-7.96)	<0.001	3.99 (1.79-8.89)	<0.001	3.81 (1.65-8.79)	0.002	8.76 (2.70-28.39)	<0.001
≥ 60	8.49 (3.89-18.52)	<0.001	8.33 (3.78-18.35)	<0.001	7.54 (3.16-18.00)	<0.001	18.82 (4.95-71.54)	<0.001
BMI≥28								
Age								
< 45	1.00 (Reference)		1.00 (Reference)		1.00 (Reference)		1.00 (Reference)	
45-60	5.52 (1.69-18.05)	0.005	5.19 (1.56-17.24)	0.007	4.74 (1.36-16.47)	0.014	11.69 (1.79-76.33)	0.010
≥ 60	5.29 (1.90-14.72)	0.001	4.38 (1.43-13.41)	0.010	3.58 (0.88-14.63)	0.075	8.47 (0.96-74.78)	0.055

Model1: Crude; Model2: Adjust: Gender, smoking, drinking and BMI; Model3: Adjust: Gender, smoking, drinking, BMI, hyptension, MASLD, heart disease and stroke; Model4: Adjust: Gender, smoking, drinking, BMI, hyptension, MASLD, heart disease, stroke, HbA1c, FBG, TC, TG, LDL, HDL, ALT, AST, Scr, UA, BUN and eGFR.

A higher BMI was found to be a protective factor in patients aged < 45 years when analysed by age group. Following full adjustment, the ORs for BMI 24–28 kg/m² and ≥ 28 kg/m² were 0.11 and 0.13 respectively (both P < 0.05). When patients were aged 45–60 years, a BMI of ≥ 28 kg/m² became a risk factor (OR = 8.30, 95% CI: 1.42-48.56, P = 0.019). For those aged ≥ 60 years, no statistically significant differences were found between the different BMI groups (all P > 0.05) (**Table 6**).

**Table 6:** Multivariate logistic regression of BMI and thyroid nodules by age stratification.

Variables	Model1		Model2		Model3		Model4	
	OR (95%CI)	*P*	OR (95%CI)	*P*	OR (95%CI)	*P*	OR (95%CI)	*P*
Age<45								
BMI								
< 24	1.00 (Reference)		1.00 (Reference)		1.00 (Reference)		1.00 (Reference)	
24-28	0.27 (0.09-0.80)	0.018	0.28 (0.09-0.81)	0.020	0.22 (0.07-0.68)	0.009	0.11 (0.02-0.55)	0.007
≥ 28	0.38 (0.13-1.10)	0.073	0.42 (0.14-1.27)	0.125	0.29 (0.08-0.97)	0.045	0.13 (0.02-0.93)	0.042
60>Age≥45								
BMI								
< 24	1.00 (Reference)		1.00 (Reference)		1.00 (Reference)		1.00 (Reference)	
24-28	0.68 (0.32-1.46)	0.322	0.77 (0.35-1.70)	0.512	0.89 (0.39-2.02)	0.775	2.38 (0.78-7.31)	0.129
≥ 28	1.42 (0.43-4.64)	0.564	2.07 (0.59-7.26)	0.257	3.15 (0.81-12.15)	0.097	8.30 (1.42-48.56)	0.019
Age≥60								
BMI								
< 24	1.00 (Reference)		1.00 (Reference)		1.00 (Reference)		1.00 (Reference)	
24-28	0.82 (0.39-1.74)	0.600	0.78 (0.36-1.68)	0.525	0.83 (0.38-1.81)	0.641	0.46 (0.17-1.24)	0.125
≥ 28	0.71 (0.26-1.94)	0.500	0.67 (0.24-1.86)	0.441	0.75 (0.26-2.21)	0.607	0.61 (0.17-2.26)	0.462

Model1: Crude; Model2: Adjust: Gender, smoking, drinking and age; Model3: Adjust: Gender, smoking, drinking, age, hyptension, MASLD, heart disease and stroke; Model4: Adjust: Gender, smoking, drinking, age, hyptension, MASLD, heart disease, stroke, HbA1c, FBG, TC, TG, LDL, HDL, ALT, AST, Scr, UA, BUN and eGFR.

## 4 Discussion

The present cross-sectional study analysed the clinical data of 720 hospitalised T2DM patients in order to investigate the prevalence of thyroid nodules and the associated risk factors. The study focused on the effects of age, BMI, and their interaction on the occurrence of thyroid nodules. The study thereby provides a reference for the screening and management of thyroid nodules in clinical practice. The results of this study indicate that, among the 720 hospitalised T2DM patients, the prevalence of thyroid nodules was 83.19%, suggesting that the occurrence of thyroid nodules is relatively common in this patient population. This finding is largely consistent with the conclusions of numerous previous studies. However, given the significant difference in sample size between the nodular and non-nodular groups, the results of the study should be interpreted with caution. It is imperative that future studies augment the sample size in order to validate these findings.

The investigation revealed that age is an independent risk factor for the development of thyroid nodules in patients with T2DM; as age increases, the risk of developing thyroid nodules increases significantly. Following full adjustment, the risk of nodule development in patients aged ≥ 60 years was 9.07 times that of patients aged < 45 years. However, BMI was identified as an independent protective factor for thyroid nodule development; an increased BMI was found to reduce the risk of nodule development, with the risk in patients with a BMI ≥28 kg/m² being only 0.46 times that of patients with a BMI < 24 kg/m². Furthermore, a significant interaction between age and BMI has been identified, with this effect being dependent on stratification. In the subgroup with a BMI of ≥ 24 kg/m², the promoting effect of age on nodule development is more pronounced, whereas in the subgroup aged < 45 years, the protective effect of BMI is more prominent; in the 45-60-year-old subgroup, a BMI of ≥ 28 kg/m² becomes a risk factor, suggesting that the two factors exert a complex synergistic effect on the development of thyroid nodules in patients with T2DM.

In this study, age was identified as an independent risk factor for thyroid nodules in patients with T2DM, consistent with the findings of several other studies. A cross-sectional study involving 896 patients with T2DM found that for every one-year increase in age, the risk of developing thyroid nodules rose by 4.8% (OR = 1.048, 95% CI: 1.023-1.074, P < 0.001), which is very close to the age-related OR value in this study (1.05, 95% CI: 1.04-1.07). The study also noted that the incidence of nodules in patients with T2DM aged ≥ 60 years was significantly higher than in those aged < 50 years, suggesting that age-related degenerative changes in thyroid function and the cumulative effects of long-term metabolic disorders may be the primary causes of this phenomenon (12). Another study demonstrated that, in age-stratified analyses, the risk of thyroid nodules was significantly elevated in T2DM patients aged 45 years and older, consistent with the trend of increased risk observed in the 45–60 and ≥ 60 age subgroups in this study. However, the study did not identify an interaction between age and BMI, which may be related to differences in sample size, geographical variations in the study populations, and the extent to which confounding factors were adjusted for (13). Controversy persists in the extant literature regarding the association between BMI and thyroid nodules in patients with T2DM.

The present study found that BMI is an independent protective factor against thyroid nodules in hospitalised T2DM patients; the risk of nodules in patients with a BMI of 24–28 kg/m² and a BMI ≥28 kg/m² was 0.52 times and 0.46 times that of those with a BMI <24 kg/m², respectively. These findings contradict the majority of conventional beliefs and several epidemiological conclusions. A plethora of extant studies on the general community population suggest that obesity may increase thyroid volume and elevate the risk of nodule development through mechanisms such as influencing thyroid hormone secretion and inducing chronic inflammation. However, other studies have shown no significant association between BMI and thyroid nodules (14, 15). It is important to note that such divergent conclusions are not isolated cases. In accordance with the study’s design and the analysis of population characteristics, the primary reasons are as follows: Firstly, there are fundamental differences in the study populations. The subjects included in this study were all hospitalised patients with T2DM. This group exhibits a more pronounced form of insulin resistance; within a certain range, an increase in body weight can improve the body’s insulin metabolism, thereby regulating the synthesis and secretion of thyroid hormones and reducing abnormal proliferation of thyroid cells. This is also the core reason why some studies on diabetic populations have similarly concluded that a high BMI has a protective effect. Secondly, it is important to note that the diagnostic criteria for nodules vary. The present study strictly excluded benign lesions, such as thyroid cysts and inflammatory nodules, including only true space-occupying nodules. In contrast, some literature does not distinguish between nodule types. It is the inconsistencies in statistical criteria that directly affect the final conclusions. Thirdly, the degree of control over confounding factors varies; different studies employ distinct adjustment strategies for confounding variables such as duration of diabetes, antidiabetic medication, liver function and blood lipid levels. Fourthly, the interaction between age and BMI is a source of confusion for the overall results; the protective effect of BMI is markedly age-dependent, and the findings for the overall population are primarily driven by data from younger subgroups.

Moreover, previous studies did not further explore the differences in the influence of age across different BMI categories. This study has addressed this research gap (16). The present study corroborates the hypothesis that there is a significant interaction between age and BMI; the combined effect of these two factors on thyroid nodules is not simply additive, and the nature of the effect changes markedly across different subgroups. The results of the subgroup analysis demonstrate that, in the overweight and obese subgroup (BMI ≥ 24 kg/m²), the role of age in promoting the development of thyroid nodules is further amplified. Among younger patients aged under 45, the protective effect of elevated BMI was most pronounced, with significantly reduced risks observed in both the BMI 24–28 kg/m² and BMI ≥28 kg/m² subgroups. In contrast, in the middle-aged group aged 45-60, a BMI of ≥28 kg/m² shifted from being a protective factor to a risk factor. It is important to note that the sample size for these cross-stratified subgroups was relatively small, resulting in extremely wide 95% confidence intervals and insufficient statistical stability. Consequently, these findings should be regarded as merely indicative of clinical trends, necessitating caution during interpretation and refraining from considering them as definitive conclusions. In the elderly population (aged ≥60 years), no statistically significant difference in nodule risk was observed across different BMI groups. This suggests that degenerative changes in the thyroid during old age become the dominant factor, to some extent masking the role of BMI. The aforementioned interaction pattern also elucidates the contradictory findings regarding the association between BMI and thyroid nodules observed in different studies. When analysing the independent association between the two alone, the effect of age-related bias is often overlooked.

With regard to other influencing factors, this study found that the incidence of thyroid nodules was significantly higher in female patients with T2DM than in male patients, which is consistent with the conclusions of most studies. The aetiology of this phenomenon is speculative, but it may be related to fluctuations in oestrogen levels in women and the higher sensitivity of thyroid tissue to hormones (17–19). In relation to the correlation between smoking, alcohol consumption and thyroid nodules, this study established that smoking and alcohol consumption function as protective factors. This finding is not consistent with the conclusions of certain studies, which have identified no significant association between smoking, alcohol consumption and thyroid nodules in patients with T2DM. Conversely, other studies have suggested that smoking may increase the risk of nodule development. This discrepancy may be attributable to variations in the definition criteria and dosage of smoking and alcohol consumption, as well as differences in the lifestyle habits of the study populations. In this study, smoking and alcohol consumption were defined as long-term, continuous exposure, which may differ from the effects of short-term exposure; further validation with a larger sample size is required (20, 21). Furthermore, the study found that a history of stroke significantly increases the risk of thyroid nodules in patients with T2DM. The paucity of literature on this topic has given rise to speculation that the phenomenon may be related to stroke-associated vascular lesions and inflammatory responses affecting thyroid tissue. However, given the cross-sectional design of the study, a definitive causal relationship between the two cannot be established; further prospective studies are required to investigate this.

It is hypothesised, based on the extant literature and the findings of this study, that the development of thyroid nodules in patients with T2DM may be associated with the following mechanisms: Firstly, the phenomenon of age-related decline in thyroid function. As individuals age, degenerative changes occur in thyroid tissue, leading to an imbalance between cell proliferation and apoptosis, which facilitates the formation of nodular lesions. Concurrently, the chronic hyperglycaemic state in T2DM patients has been demonstrated to exacerbate oxidative stress damage to thyroid tissue, thereby accelerating nodule formation. Secondly, the protective effect of BMI against thyroid nodules may be related to insulin resistance. A higher BMI has been shown to enhance the synthesis and secretion of thyroid hormones by regulating insulin levels, thereby reducing abnormal proliferation of thyroid cells. However, in middle-aged patients, a BMI ≥ 28 kg/m² becomes a risk factor, which may be due to obesity-related chronic inflammatory responses outweighing its protective effects. Thirdly, the interaction between age and BMI may stem from differences in the sensitivity of thyroid tissue to metabolic disturbances across different age groups. The protective effect of BMI is more pronounced in younger patients, whereas in older patients, age-related degenerative changes predominate, masking the influence of BMI (22–25).

The present study is subject to several limitations. Firstly, given that this is a cross-sectional study, it can only establish the association between various factors and thyroid nodules; it cannot infer a causal relationship. Secondly, this is a single-centre study that included only inpatients in the Endocrinology Department. Given the propensity of this demographic to exhibit more severe symptoms and a higher prevalence of comorbidities, there is considerable selection bias. The study’s findings are therefore applicable exclusively to inpatients with T2DM at this centre and cannot be directly extrapolated to the general community, outpatients with T2DM, or similar populations in other regions. Thirdly, the distribution of sample sizes was found to be uneven across the groups. The sample size in the thyroid nodule group was found to be significantly larger than that in the nodule-free group, and the sample sizes in some cross-stratified subgroups were found to be small. This resulted in wide confidence intervals for certain indicators and reduced the stability of the statistical results. Finally, the study did not collect detailed data on the size, number, histological classification, or thyroid function of the thyroid nodules. This precluded further analysis of the association between various risk factors and nodule characteristics. Furthermore, potential confounding factors such as genetics and dietary habits were not included, which may have introduced some bias into the results.

## 5 Conclusion

The present study demonstrates that both age and BMI independently influence the risk of thyroid nodules in hospitalised T2DM patients, and that there is a significant interaction between the two. Advanced age has been identified as a risk factor, whilst a high BMI has generally been shown to have a protective effect. However, the risk is higher in older patients who are overweight or obese. It is important to note that the findings of this study are applicable only to hospitalised T2DM patients at a single centre and should not be extrapolated indiscriminately.

## Data Availability

The raw data supporting the conclusions of this article will be made available by the authors, without undue reservation.
